# Sub-Inhibitory Concentrations of Metronidazole Enhance Production, Virulence Factor Loading, and Endothelial Cytotoxicity of *Porphyromonas gingivalis* Extracellular Vesicles

**DOI:** 10.3390/microorganisms14010025

**Published:** 2025-12-21

**Authors:** Zixiang Wu, Xia Li, Song Ge

**Affiliations:** School of Stomatology, Zunyi Medical University, Zunyi 563000, China; wuzixiang521@foxmail.com (Z.W.); lixia711963@foxmail.com (X.L.)

**Keywords:** *Porphyromonas gingivalis*, extracellular vesicles, atherosclerosis, periodontitis, metronidazole, human umbilical vein endothelial cells

## Abstract

*Porphyromonas gingivalis* (*P. gingivalis*), a key periodontal pathogen, has been linked to atherosclerosis development. The clinical failure of antibiotics to improve cardiovascular outcomes necessitates alternative explanations. This study examines how sub-inhibitory concentrations of metronidazole affect the biogenesis and pathogenic potential of *P. gingivalis* extracellular vesicles (EVs) on human umbilical vein endothelial cells (HUVECs). EVs were isolated from both untreated bacteria (N-EVs) and those treated with sub-inhibitory concentrations of metronidazole (M-EVs) through ultracentrifugation. Characterization included transmission electron microscopy (TEM), nanoparticle tracking analysis, and Western blotting for virulence factors. HUVECs were evaluated using viability, migration, cell death assays, ROS detection, NF-κB activation imaging, and cytokine measurement. Sub-inhibitory concentrations of metronidazole increased EV production by 2.3-fold and enriched M-EVs with virulence factors (lipid A LPS, Kgp, RgpA). M-EVs demonstrated significantly stronger cytotoxicity, causing greater impairment of HUVEC viability and migration, alongside increased cell death. Mechanistically, M-EVs induced elevated mitochondrial and cellular ROS, promoting NF-κB activation and enhancing secretion of pro-inflammatory cytokines (TNF-α, IL-1β, IL-6). Sub-inhibitory concentrations of metronidazole exacerbate endothelial injury by amplifying EV production and virulence factor loading in *P. gingivalis*, offering a mechanistic explanation for the limited cardiovascular benefits of antibiotic therapy in periodontitis patients.

## 1. Introduction

Periodontitis is an inflammatory condition affecting the supporting structures of the teeth and represents one of the most prevalent chronic infectious diseases in humans [1]. This disease results in the destruction of periodontal connective tissue and resorption of the alveolar bone, constituting the primary cause of tooth loss in adults. Inflammation of the periodontal tissues can lead to localized tissue damage, ulceration, and bleeding within the lining of periodontal pockets [1,2]. In cases of severe, extensive periodontitis, the total ulcerated area on the inner walls of periodontal pockets may reach approximately 72 cm^2^, which is comparable to the surface area of an adult human palm. This extensive ulceration facilitates transient but recurrent bacteremia, which can be induced by routine oral hygiene practices, periodontal treatments, and even mastication [3,4]. Consequently, periodontal pathogens such as *Porphyromonas gingivalis* (*P. gingivalis*) and their virulence factors can enter the bloodstream, potentially compromising the integrity and function of endothelial cells and exerting effects on vascular smooth muscle cells [2].

Numerous ex vivo and in vivo studies have demonstrated that infection with *P. gingivalis* induces the transformation of mouse macrophages into foam cells. Repeated bacteremia caused by *P. gingivalis* has been shown to induce or exacerbate atherosclerosis (As)-like lesions in the aorta and coronary arteries of experimental pigs [4,5]. Furthermore, *P. gingivalis* oral infection accelerates the progression of early atherosclerotic lesions in apolipoprotein E knockout (ApoE-/-) mice [4]. However, despite numerous studies suggesting a link between *P. gingivalis* infection and the development of As and the frequent detection of bacterial biomolecules such as nucleic acids within atherosclerotic plaques, live bacteria are rarely isolated from these lesions [6]. Moreover, some clinical studies have reported that antibiotic treatment does not significantly reduce vascular wall inflammation or subsequent adverse cardiovascular events in patients with periodontitis and cardiovascular disease (CVD) [7,8]. This paradoxical observation raises important questions regarding the hypothesis that *P. gingivalis* influences the progression of As and the association between periodontitis and As.

Gram-negative bacteria continuously produce and secrete extracellular vesicles (EVs) during their growth [9]. Among the principal periodontal pathogens, *P. gingivalis* is recognized as a particularly prolific producer of EVs. These EVs are double-membrane vesicles ranging from approximately 20 to 250 nm in diameter and are enriched with key virulence factors, including lipopolysaccharide (LPS) and gingipains. Notably, *P. gingivalis* EVs exhibit greater stability and pathogenic potential compared to their parent bacterial cells [9]. Functionally, *P. gingivalis* EVs contribute to adhesion and colonization, host cell invasion, biofilm formation, host cell damage, and modulation of host immune responses, often exerting effects comparable to or exceeding those of the parental bacteria. Furthermore, studies have demonstrated that *P. gingivalis* EVs can disseminate via the bloodstream to distant organs such as the heart, liver, lungs, and spleen, where they are implicated in the pathogenesis of various systemic diseases [10].

Although the biogenesis of *P. gingivalis* EVs has not been fully elucidated, various factors have been demonstrated to influence their production and yield [9,10]. Recent studies have shown that antibiotic-induced stress not only enhances the secretion of bacterial EVs, but also significantly modifies their pathogenic characteristics. For instance, Bauwens et al. [11] reported that ciprofloxacin treatment increased the production of enterohemorrhagic *Escherichia coli* (*E. coli*) EVs and concurrently upregulated the levels of its principal virulence factor, Shiga toxin 2a, potentially exacerbating the severity of clinically relevant infections.

In summary, the pathogenic effects and mechanisms of bacterial EVs produced under antibiotic-induced stress conditions may differ from those generated under conventional conditions. To date, the influence of antibiotics—particularly those commonly employed in periodontal therapy—on the biogenesis of *P. gingivalis* EVs and their pathogenic characteristics remains unclear. In the non-surgical treatment of periodontitis, the application of local and systemic antimicrobial agents serves as a key adjunctive measure. Commonly used clinical drugs include amoxicillin, doxycycline, metronidazole, and various compound preparations [12]. Among these, metronidazole is particularly widely used in clinical practice due to its highly targeted antimicrobial activity against obligate anaerobic bacteria such as *P. gingivalis* [12]. Therefore, the present study aims to investigate the impact of sub-inhibitory concentrations of metronidazole on the biogenesis of *P. gingivalis* EVs and to evaluate whether these EVs enhance cytotoxicity, induce oxidative stress, and exacerbate inflammatory responses in endothelial cells. This investigation seeks to provide a foundation for elucidating the underlying mechanisms contributing to the limited clinical efficacy of antibiotics in the treatment of periodontitis-associated CVD.

## 2. Materials and Methods

### 2.1. Bacterial Culture

*P. gingivalis* (strain ATCC 33277) was purchased from BeiNa Biological Company (BNCC, Beijing, China) and initially cultured on blood agar plates at 37 °C under anaerobic conditions (80% N_2_, 10% CO_2_, 10% H_2_) for 6–8 days to reactivate the strain. Subsequently, the activated bacterial strains were inoculated at 5% (*v*/*v*) into Tryptic Soy Broth (TSB; Cat No: LA0360, Solarbio, Beijing, China) liquid medium supplemented with 0.5% hemin chloride (Cat No: H8132, Solarbio) and 0.1% vitamin K_1_ (Cat No: V8151, Solarbio). The cultures were incubated anaerobically in a shaking incubator at 37 °C until the late logarithmic growth phase (OD_600_ ≈ 1.0) was reached (Supplementary Figure S1B). The bacterial suspension was then centrifuged (1000× *g*, 5 min), and the resulting pellet was resuspended in fresh medium and adjusted to a concentration of 1 × 10^6^ CFU/mL for subsequent use.

Based on the clinical application profile of metronidazole and the objectives of the experimental design, a concentration of 2 μg/mL was selected for investigation as a sub-inhibitory concentration. This concentration approximates the drug level found in gingival tissues following oral metronidazole administration for periodontitis treatment [13], thereby simulating the local drug microenvironment achieved in clinical practice and holding translational relevance. Concurrently, preliminary experiments verified that at 2 μg/mL metronidazole, *P. gingivalis* maintained stable growth (Table S1), while a significant difference in EV yield was observed (Supplementary Figure S2B). This concentration effectively supports the core research aims. Furthermore, being substantially lower than the determined MIC of 64 μg/mL for the strain, 2 μg/mL unequivocally falls within the defined range of sub-inhibitory concentrations.

### 2.2. Transmission Electron Microscopy Observations

*P. gingivalis* bacteria, with or without 2 μg/mL metronidazole treatment for 30 min, were fixed with 2.5% glutaraldehyde at 4 °C for 24 h. Subsequently, the bacterial pellets were collected by centrifugation at 3000× *g* for 20 min and fixed again with fresh 2.5% glutaraldehyde for another 24 h. After post-fixation with 2% osmium tetroxide for 2 h, the samples were dehydrated through a graded ethanol series (30%, 50%, 70%, 85%, 95%, 100%, and 100%), dried in acetone (three changes, 15 min each), and embedded in resin blocks. The blocks were sectioned into ultrathin (70 nm) slices, which were stained with uranyl acetate and lead citrate [14]. The prepared samples were observed using a JEM-1400FLASH transmission electron microscopy (TEM; JEOL, Tokyo, Japan).

### 2.3. Scanning Electron Microscope Observation

Sterile glass coverslips (φ = 24 mm) were placed in 6-well plates. Activated bacterial suspension at 1 × 10^6^ CFU/mL was added at 2 mL per well and incubated anaerobically for 2 h. Metronidazole solution dissolved in dimethyl sulfoxide (DMSO: Cat No: D8372, Solarbio) was then added to each well to achieve a final concentration of 2 μg/mL, with an equal volume of DMSO used as a blank control. After 30 min of anaerobic incubation at 37 °C, the bacteria were fixed with 2.5% glutaraldehyde at 4 °C for 24 h. In addition, 200 μL aliquots of *P. gingivalis* cultures, either treated or untreated with 2 μg/mL metronidazole for 54 h (the late logarithmic growth phase) (Supplementary Figure S1B) under anaerobic conditions at 37 °C, were transferred onto sterile glass coverslips (φ = 24 mm) placed in 6-well plates. After incubation at room temperature for 1 h, the samples were fixed with 2.5% glutaraldehyde at 4 °C for 24 h. For all samples, the fixative was carefully aspirated, and the coverslips were washed by immersion in phosphate-buffered saline (PBS: Cat No: P1020, pH 7.2–7.4, Solarbio) buffer for 10 min (repeated three times). After PBS removal, dehydration was performed sequentially using 50%, 70%, and 90% ethanol (10 min each), followed by two changes in absolute ethanol (10 min each). The samples were then treated with an ethanol–pure tert-butanol mixture (1:1, *v*/*v*) for 15 min, followed by two changes in pure tert-butanol (15 min each). After tert-butanol removal, the samples were freeze-dried and sputter-coated with gold [15]. Observation and imaging were performed using an SU8010 scanning electron microscope (SEM; Hitachi, Tokyo, Japan).

### 2.4. Isolation and Identification of P. gingivalis EVs

Activated bacterial suspension at 1 × 10^6^ CFU/mL was inoculated into 500 mL of fresh TSB liquid medium at a 1:10 (*v*/*v*) ratio. The cultures were then randomly allocated to two groups: the control group received an equal volume of DMSO solvent, while the metronidazole-treated group received a metronidazole solution (dissolved in DMSO) at the beginning of cultivation, yielding a final concentration of 2 μg/mL. Both groups were cultured under anaerobic conditions in a shaking incubator at 37 °C for 54 h. *P. gingivalis* EVs were isolated according to a previously described protocol with modifications [16]. After cultivation, the medium was immediately centrifuged at 4 °C and 8000× *g* for 30 min to remove the majority of bacterial cells. The resulting supernatant was collected and filtered through a 0.22 μm sterile filter to remove residual bacteria and debris. The filtrate was then concentrated using a 100 kDa ultrafiltration unit centrifuged at 4 °C, 2500× *g* for 30 min. The concentrate was transferred to a 30 mL ultracentrifuge tube and subjected to ultracentrifugation at 4 °C, 100,000× *g* for 80 min. The pellet was resuspended in a small volume of sterile PBS and filtered again through a 0.22 μm filter to remove larger vesicles and residual debris. A second round of ultracentrifugation was performed under the same conditions (4 °C, 100,000× *g*, 80 min). The final pellet was resuspended in 1.5 mL of sterile PBS, yielding naturally derived EVs (N-EVs) and sub-inhibitory concentrations of metronidazole-treated derived EVs (M-EVs), which were stored at −80 °C for subsequent use.

For TEM characterization, 10 μL of N/M-EVs sample was applied onto a copper grid, allowed to settle for 1 min, and the excess liquid was blotted away with filter paper. Then, 10 μL of uranyl acetate (Cat No: 1722586, Beijing Zhongjing Keqi Technology Co., Ltd., Beijing, China) was applied to the grid for 1 min, and the excess was blotted off. After air-drying at room temperature for several min, the samples were imaged using an HT7700 TEM (Hitachi, Tokyo, Japan). For nanoparticle characterization, 10 μL of N/M-EVs were each diluted to an appropriate concentration and analyzed using an N30E Nanoflow cytometer (NanoFCM, Xiamen, China). The instrument acquired and analyzed scattered light signals from individual particles to determine the EVs’ size distribution, average particle size, and concentration.

### 2.5. Protein Concentration Measurement

The protein concentration of the extracted *P. gingivalis* EVs was quantified using a bicinchoninic acid (BCA) protein assay kit (Cat No: PC0021, Solarbio) according to the manufacturer’s instructions.

### 2.6. Immunoblotting

EVs samples were lysed using 80 μL of exosomal protein lysis buffer (Cat No: IL9020, Solarbio). Protein concentration was determined using the BCA protein assay kit as per the manufacturer’s protocol. For Western blotting, equal amounts of protein were loaded onto SDS-PAGE gels and subsequently transferred onto polyvinylidene fluoride (PVDF) membranes (Cat No: IPVH00010, Millipore, Darmstadt, Germany). The membranes were blocked with 5% Bovine Serum Albumin (BSA; Cat. No.: SW3015, Solarbio) for 30 min at room temperature. They were then incubated overnight at 4 °C with the following primary antibodies: lipid A LPS (Cat No: NB100-64484, Novus Biologicals, Littleton, CO, USA), Kgp (Cat No: abx338983, Abbexa, Cambridge, UK), and RgpA (Cat No: LS-C371047, LSBio, Seattle, WA, USA). Following primary antibody incubation, the membranes were incubated with corresponding species-specific secondary antibodies for 30 min at room temperature with shaking. Enhanced chemiluminescence (ECL) reagent was prepared by mixing the two components in a 1:1 ratio. The PVDF membranes were exposed to the ECL reagent, and images were automatically captured using a protein imaging system.

### 2.7. Cell Culture

HUVECs (Cat No: DFSC-EC-01, Shanghai ZQXZBIO Biotechnology Co., Ltd., Shanghai, China) were cultured in a specialized medium (Cat No:PCM-H-040, ZQXZBIO) according to the manufacturer’s instructions. The cells were cultured at 37 °C in a humidified atmosphere containing 5% CO_2_. Upon reaching approximately 80% confluence, HUVECs were passaged and employed for experimental procedures between the third and fifth passages.

### 2.8. Endocytosis Analysis

The lipid membranes of *P. gingivalis* EVs were labeled using the PKH26 red fluorescent cell linker kit (Cat No: UR52302, Umibio (Shanghai) Co., Ltd., Shanghai, China). Briefly, the extracted N-EVs and M-EVs were incubated with 1 μL PKH26 dye and 9 μL Diluent C in 100 μL PBS for 15 min (PBS buffer alone served as the control). Subsequently, 1 mL of PBS was added, and the mixture was centrifuged at 4 °C, 100,000× *g* for 2 h. The supernatant was discarded, and the pellet was resuspended in sterile PBS. HUVECs were then incubated with the PKH26-labeled N-EVs or M-EVs at 37 °C for 24 h. After incubation, the cells were fixed with 4% paraformaldehyde for 15 min. Images were acquired using a Ti-s inverted fluorescence microscope (Nikon, Tokyo, Japan).

### 2.9. Cell Viability Analysis

Cell viability of HUVECs was assessed using the Cell Counting Kit-8 (CCK-8; Cat No: K1018, APExBIO, Houston, TX, USA). HUVECs were seeded in 96-well plates at a density of 1 × 10^4^ cells per well. After treatment with *P. gingivalis* EVs at various concentrations (0, 5, 10, 20, 50, and 100 μg/mL) for 24 h, 10 μL of CCK-8 solution was added to each well. The plate was incubated for 24 h, and the optical density (OD) at 450 nm was measured. Cell viability (%) was calculated as follows: [(OD experiment − OD control)/(OD control − OD blank)] × 100%.

### 2.10. Cell Morphology Analysis

HUVECs were seeded directly into 12-well plates at a density of 1 × 10^5^ cells per well and incubated overnight at 37 °C with 5% CO_2_. The cells were then treated with 20 μg/mL of either N-EVs or M-EVs for 24 h. After treatment, the old medium was removed, and the cells were gently washed three times with PBS. Fresh medium (1 mL) was added to each well, and changes in cell morphology were observed using an inverted optical microscope.

### 2.11. Cell Migration Capacity Analysis

HUVECs were seeded in 6-well plates at a density of 2 × 10^5^ cells per well. When the cells reached 80–90% confluence, a straight scratch was created using a 1000 μL pipette tip. The dislodged cells were washed away with PBS. An appropriate amount of Hoechst 33342 (Cat No: C1025, Shanghai Beyotime Biotechnology Co., Ltd., Shanghai, China) was added to stain the nuclei, incubating for 5 min. Images of the scratch center were captured at 0 h using an inverted fluorescence microscope. After treatment with 20 μg/mL of N-EVs or M-EVs for 24 and 48 h, respectively, the same staining and imaging procedures were repeated to capture images at the respective time points. The scratch area was measured and analyzed using ImageJ software.

### 2.12. Propidium Iodide Staining

The number of late apoptotic cells was detected using the Propidium Iodide (PI) Apoptosis Detection Kit (Cat No: C1352S, Beyotime) according to the manufacturer’s instructions. HUVECs were seeded on coverslips (1 × 1.0 cm) placed in 12-well plates at a density of 1 × 10^5^ cells per well and incubated overnight at 37 °C with 5% CO_2_. After treatment with 20 μg/mL of N-EVs or M-EVs for 24 h, the old medium was carefully aspirated. Without washing with PBS, 400 μL of binding buffer and 10 μL of PI staining solution were directly added to each well, followed by incubation in the dark at room temperature for 10 min. Subsequently, an appropriate amount of Hoechst 33342 was added and incubated for 5 min to stain all nuclei. Images were acquired directly using an inverted fluorescence microscope.

### 2.13. Reactive Oxygen Species Analysis

The levels of mitochondrial reactive oxygen species (mtROS) and total cellular reactive oxygen species (ROS) in HUVECs treated with 20 μg/mL of N-EVs or M-EVs for 24 h were detected using Mito-SOX Red mitochondrial superoxide indicator (Cat No: BB25021, BestBio, Xi’an, China) and 2′,7′-dichlorodihydrofluorescein diacetate (DCFH-DA; Cat No: S0033S, Beyotime) probes, respectively, following the manufacturers’ protocols. HUVECs were seeded in 12-well plates at a density of 1 × 10^5^ cells per well and incubated overnight at 37 °C with 5% CO_2_. After respective treatments for 24 h, the old medium was removed, and the cells were washed with PBS. Pre-warmed (37 °C) Mito-SOX Red working solution (prepared at a ratio of Mito-SOX Red fluorescent probe: probe diluent: medium = 1:10:200) was added, and the cells were incubated under growth conditions in the dark for 15 min. The cells were then washed with PBS. Subsequently, an appropriate amount of DCFH-DA probe was added and incubated in the dark for 30 min. After three washes with PBS, Hoechst 33342 was added and incubated for 5 min to stain nuclei. Following three additional PBS washes, 2 mL of fresh, pre-warmed (37 °C) medium was added. Images were captured using an inverted fluorescence microscope, and fluorescence intensity was analyzed using ImageJ V1.54g.

### 2.14. Immunofluorescence Staining

HUVECs were seeded at a density of 2 × 10^4^ cells per well in 48-well plates. Following 24 h of the designated treatment, the cells were fixed with 4% paraformaldehyde for 10 min, washed three times with PBS, and subsequently permeabilized with immunostaining permeabilizing solution containing Triton X-100 (Cat No: P0096, Beyotime) for 10 min at room temperature. The cells were then blocked with 5% bovine serum albumin (BSA) blocking solution (Cat No: SW3015, Solarbio) for 1 h at room temperature. Primary antibodies against nuclear factor-kappa B (NF-κB) p65 (1:100, Cat No: AB2020, Beyotime, China) was applied and incubated overnight at 4 °C. After removal of the primary antibodies and three washes with PBS, the cells were incubated with the secondary antibody (1:1000, Cat No: AF350-labeled goat anti-rabbit IgG, Beyotime) for 1 h at room temperature. Following three additional PBS washes, cell nuclei were counterstained with Hoechst 33342. IF images were captured using an inverted fluorescence microscope, and fluorescence intensity was quantified using ImageJ software.

### 2.15. Enzyme-Linked Immunosorbent Assay Detection

The cell culture supernatants from each group were collected into 1.5 mL microcentrifuge tubes and centrifuged at 350× *g* for 20 min at 2–8 °C to remove cell debris and impurities. The resulting clarified supernatants were collected. The expression levels of tumor necrosis factor-alpha (TNF-α; Cat No: HTQ-ES-00090, Changzhou Haotianqi Biotechnology Co., Ltd. (Mostcell), Changzhou, China), interleukin-1 beta (IL-1β; Cat No: HTQ-ES-00140, Mostcell), and interleukin-6 (IL-6; Cat No: HTQ-ES-00035, Mostcell) were measured using respective commercial ELISA kits (Mostcell, Changzhou, China) according to the manufacturers’ instructions.

### 2.16. Statistical Analysis

All experiments were conducted in triplicate or more, and the resulting data were analyzed and visualized using GraphPad Prism 9.0.0 (GraphPad Software, San Diego, CA, USA). Statistical significance was assessed using unpaired *t*-tests or ANOVA. The results are presented as the mean  ±  standard deviation (SD). A *p* value < 0.05 was considered significant.

## 3. Results

### 3.1. Sub-Inhibitory Concentrations of Metronidazole Modulate P. gingivalis EV Production

**Figure 1:**
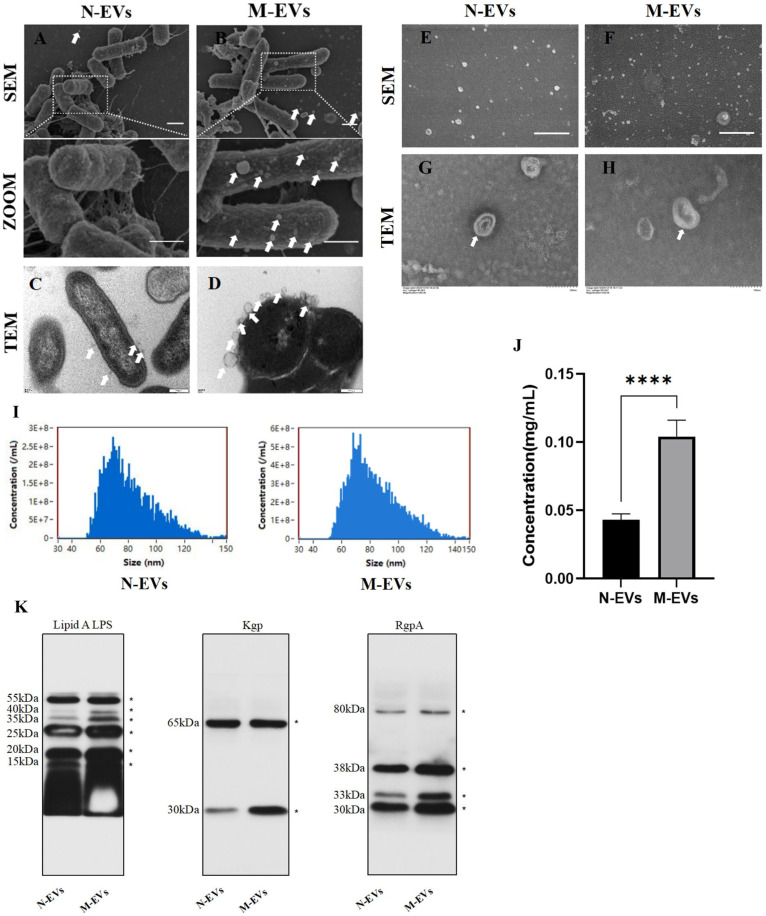
Sub-inhibitory concentrations of metronidazole induce enhanced biogenesis and virulence factor loading in *P. gingivalis* EVs. (**A**,**B**) SEM images showing the surface morphology of *P. gingivalis* (scale bar, 1 µm); The white arrow indicates EVs. (**C**,**D**) TEM images illustrating EVs budding from the outer membrane of *P. gingivalis* (scale bar, 200 nm); The white arrow indicates EVs. (**E**,**F**) SEM images of EVs present in the supernatant of *P. gingivalis* cultures (scale bar, 1 µm); The white arrow indicates EVs. (**G**,**H**) TEM images of N/M-EVs isolated by ultracentrifugation (scale bar, 100 nm); The white arrow indicates EVs. (**I**) Size distribution profiles of N/M-EVs as determined by NTA. (**J**) Protein concentration of N/M-EVs quantified by BCA assay. (**K**) Western blotting analysis of lipid A, Kgp, and RgpA protein levels in N/M-EVs; * indicates molecular weight. Data in panel (**J**) were analyzed by an unpaired *t*-test and are presented as the mean ± SD from three independent experiments. Statistical significance is indicated as follows: **** *p* < 0.0001.

To determine whether sub-inhibitory concentrations of metronidazole affect the production of *P. gingivalis* EVs, *P. gingivalis* was co-cultured with 2 μg/mL metronidazole for 30 min. The number of EVs on the bacterial outer membrane was observed using SEM and TEM. The results revealed that, compared to the blank control group, sub-inhibitory concentrations of metronidazole stress within a short period induced greater production of EVs on the outer membrane of *P. gingivalis* (Figure 1A–D). Similarly, in the supernatant collected at the late logarithmic growth phase, the quantity of *P. gingivalis* EVs secreted under sub-inhibitory concentrations of metronidazole culture conditions was higher than that in the blank control group (Figure 1E,F). Subsequently, N-EVs and M-EVs isolated via classical ultracentrifugation were characterized by TEM (Figure 1G,H) and nanoparticle tracking analysis (NTA). NTA revealed that the average diameter of M-EVs (83.4 ± 17.6 nm) was slightly larger than that of N-EVs (81.8 ± 17.8 nm). The particle concentration of M-EVs (3.14 × 10^10^ particles/mL) was approximately 2.24 times higher than that of N-EVs (1.40 × 10^10^ particles/mL) (Figure 1I). BCA protein assay showed that the protein concentration of M-EVs (0.1042 ± 0.0113 mg/mL) was about 2.4 times greater than that of N-EVs (0.0431 ± 0.0041 mg/mL) (Figure 1J). However, pre-experimental results demonstrated that the total yield of EVs obtained at a metronidazole concentration of 4 μg/mL showed no statistically significant difference compared to that of N-EVs (Supplementary Figure S2B). The likely explanation for this observation is that, although EV production efficiency per bacterium may have been elevated at this concentration, the pronounced growth inhibition caused by 4 μg/mL metronidazole significantly reduced the total bacterial population. The combined effect of these two factors presumably resulted in no net change in overall EVs yield. This interpretation is corroborated at the morphological level: SEM revealed substantial alterations and damage to the cell morphology and surface structure of *P. gingivalis* following treatment with 4 μg/mL metronidazole (Supplementary Figure S2C). Such subcellular damage likely directly compromised normal bacterial growth and proliferation, thereby fundamentally limiting the total number of bacteria available for EVs production.

Our Coomassie brilliant blue staining results demonstrated that the protein bands of *P. gingivalis* whole-cell lysates covered the full range of 25–250 kDa. In contrast, *P. gingivalis* EVs extracted from this bacterium showed only a specific band at 80–90 kDa, with no detection of the broad-spectrum miscellaneous bands characteristic of total bacterial proteins. This indicates that the EVs extraction procedure effectively removed the majority of contaminating bacterial protein impurities (Supplementary Figure S1C). Recent studies have indicated that lipid A and gingipains are not only major virulence factors carried by EVs but also participate in influencing EV biogenesis [9,17]. Western blot analysis demonstrated that the protein bands for lipid A, Kgp, and RgpA at multiple molecular weights were broader in M-EVs compared to N-EVs (Figure 1K), suggesting that M-EVs carry larger quantities of lipid A, Kgp, and RgpA than N-EVs. Further analysis revealed that both Kgp in N-EVs and M-EVs exhibited two specific bands at 65 kDa and 30 kDa, while RgpA was present in four molecular forms: 80 kDa, 38 kDa, 33 kDa, and 30 kDa. This phenomenon is presumed to be associated with post-translational processing and modification, domain cleavage, and maturation of gingipains, consistent with their multi-isoform expression pattern. These results confirm that the target gingipains stably and completely exist in the extracted outer membrane vesicles, retaining the typical features of pathogen-associated molecular patterns [18,19].

In conclusion, these findings collectively indicate that sub-inhibitory concentrations of metronidazole promote the production of *P. gingivalis* EVs and enhances their loading of virulence factors.

### 3.2. Sub-Inhibitory Concentrations of Metronidazole-Derived P. gingivalis EVs Potentiate Cytotoxic Effects of HUVECs

**Figure 2:**
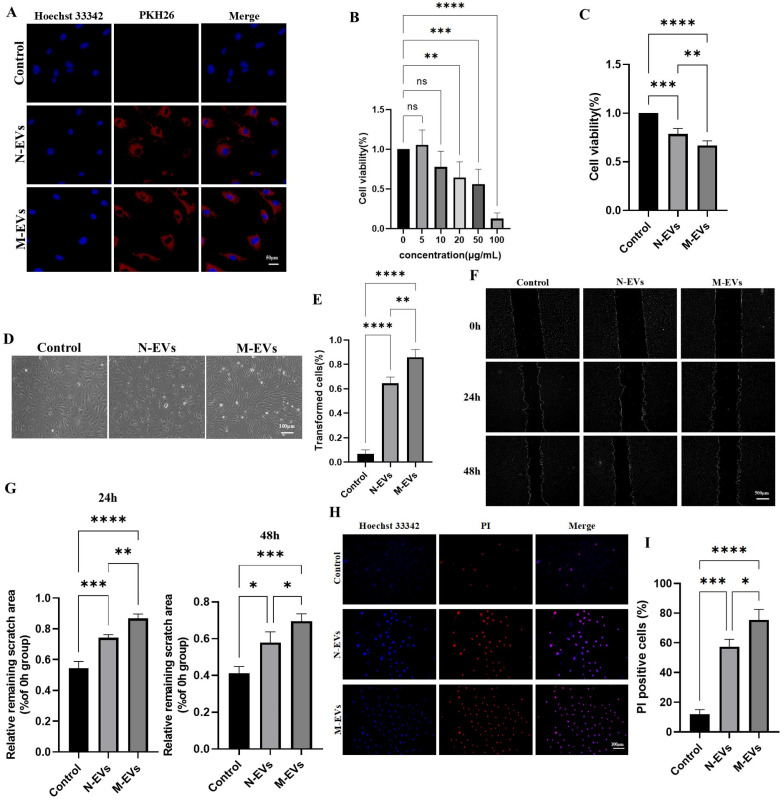
*P. gingivalis* EVs derived under sub-inhibitory concentrations of metronidazole stress exhibit enhanced cytotoxicity toward HUVECs. (**A**) Internalization of PKH26-labeled EVs (red) by HUVECs detected by fluorescence microscopy; nuclei were stained with Hoechst 33342 (blue) (scale bar, 50 μm). (**B**,**C**) Cell viability assessed by CCK-8 assay. (**D**) Morphological changes in HUVECs observed under an inverted optical microscope. (**E**) Quantitative analysis of (**D**). (**F**) Cell migration ability detected by wound healing assay; nuclei were stained with Hoechst 33342 (blue); images were processed using ImageJ (scale bar, 500 μm). (**G**) Quantitative analysis of (**F**). (**H**) PI staining (red) of HUVECs; nuclei were stained with Hoechst 33342 (blue) (scale bar, 100 μm). (**I**) Quantitative analysis of (**G**). Data in panels (**B**,**C**,**F**,**H**) were analyzed by one-way ANOVA and are presented as the mean ± SD from three independent experiments. Statistical significance is indicated as follows: ns = not significant; * *p* < 0.05; ** *p* < 0.01; *** *p* < 0.001; **** *p* < 0.0001.

The vascular endothelium, the outermost layer between the blood and the arterial intima, experiences dysfunction as an initiating factor in atherosclerosis [20]. We assessed whether *P. gingivalis* EVs exhibit cytotoxicity towards HUVECs and whether EVs derived from *P. gingivalis* under sub-inhibitory concentrations of metronidazole stress could potentiate this toxic effect. Fluorescent tracing experiments initially revealed that both N-EVs and M-EVs could be internalized by HUVECs (Figure 2A), suggesting a potential mechanism whereby *P. gingivalis* EVs deliver virulence factors intracellularly to HUVECs, possibly inducing cytopathic alterations. CCK-8 assays demonstrated that *P. gingivalis* EVs inhibited HUVECs viability in a concentration-dependent manner, with a significant effect observed at 20 µg/mL (Figure 2B). Based on this finding, 20 µg/mL was established as the minimum biological effect concentration and served as the standard treatment condition for subsequent experiments. Comparing the effects of 20 µg/mL N-EVs and M-EVs revealed that M-EVs exerted a significantly stronger inhibitory effect on HUVEC viability than N-EVs (Figure 2C). Morphological observation indicated that M-EVs induced a more pronounced phenotypic shift in HUVECs, from the typical cobblestone morphology to polygonal, fibroblast-like, or elongated spindle-shaped cells (Figure 2D,E). The wound healing assay confirmed that both N-EVs and M-EVs significantly inhibited HUVEC migration, with M-EVs exhibiting a markedly greater inhibitory effect (Figure 2F,G). PI staining results further demonstrated that M-EVs possessed a stronger capacity to induce HUVEC death (Figure 2H,I). Collectively, these findings indicate that *P. gingivalis* EVs exert substantial cytotoxicity on HUVECs, and EVs derived under sub-inhibitory concentrations of metronidazole stress exhibit an enhanced cytotoxic effect.

### 3.3. Sub-Inhibitory Concentrations of Metronidazole-Derived P. gingivalis EVs Potentiate ROS Generation in HUVECs

**Figure 3:**
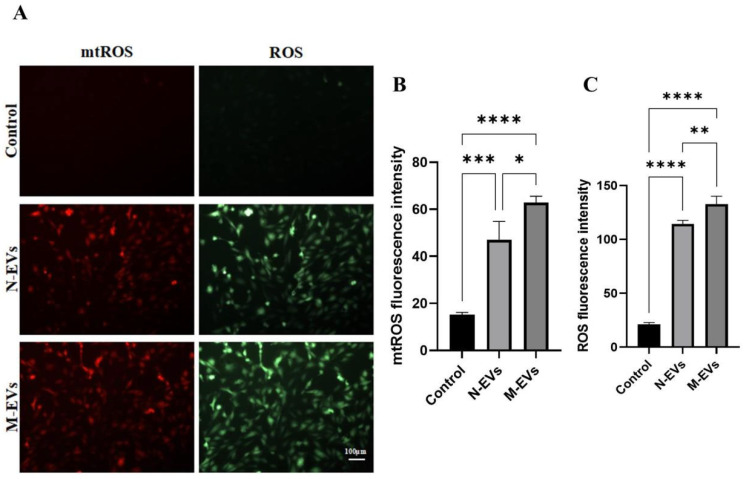
*P. gingivalis* EVs derived under sub-inhibitory concentrations of metronidazole stress exacerbate oxidative stress in HUVECs. (**A**) Levels of mtROS (red) and total cellular ROS (green) in HUVECs following treatment with N/M-EVs for 24 h. (**B**) Quantitative analysis of mtROS levels. (**C**) Quantitative analysis of total cellular ROS levels. Data in panels (**B**,**C**) were analyzed by one-way ANOVA and are presented as the mean ± SD from three independent experiments. Statistical significance is indicated as follows: * *p* < 0.05; ** *p* < 0.01; *** *p* < 0.001; **** *p* < 0.0001.

Excessive generation of ROS is a key driver of oxidative stress and dysfunction in endothelial cells [21]. To determine whether N-EVs and M-EVs could promote the burst generation of ROS in HUVECs, we intervened HUVECs with N-EVs and M-EVs at a concentration of 20 μg/mL for 24 h. We used specific fluorescent probe staining to detect the levels of mtROS and total cellular ROS generation. The results showed that both N-EVs and M-EVs significantly promoted the generation of mtROS and total cellular ROS, and the level of mtROS and total cellular ROS generation under the treatment of M-EVs was significantly higher than that of the effect of N-EVs (Figure 3A–C), suggesting that *P. gingivalis* EVs have the role of inducing oxidative stress in HUVECs and that *P. gingivalis* EVs produced by *P. gingivalis* in metronidazole incubation can enhance the oxidative stress response of HUVECs.

### 3.4. Sub-Inhibitory Concentrations of Metronidazole Potentiates P. gingivalis EV-Mediated Pro-Inflammatory Response in HUVECs

**Figure 4:**
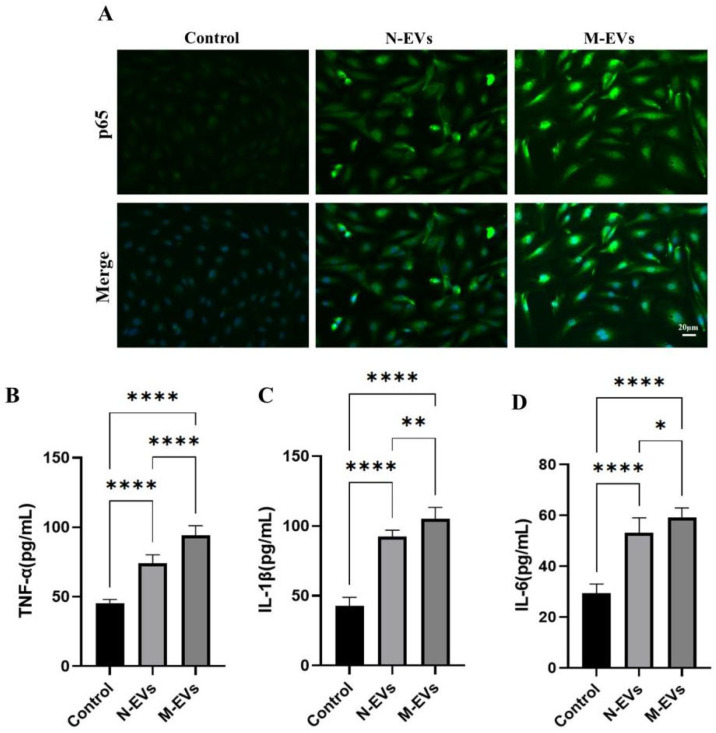
*P. gingivalis* EVs derived under sub-inhibitory concentrations of metronidazole stress potentiate the inflammatory response in HUVECs. (**A**) IF analysis of nuclear translocation of the NF-κB p65 subunit (green); nuclei were stained with Hoechst 33342 (blue). (**B**–**D**) Secretion levels of TNF-α, IL-1β, and IL-6 in culture supernatants were measured by ELISA. Data in panels (**B**–**D**) were analyzed by one-way ANOVA and are presented as the mean ± SD from three independent experiments. Statistical significance is indicated as follows: * *p* < 0.05; ** *p* < 0.01; **** *p* < 0.0001.

Previous studies have demonstrated that ROS act as signaling molecules that activate the NF-κB signaling pathway in endothelial cells, thereby promoting endothelial inflammation and exacerbating endothelial dysfunction [22,23]. To investigate whether the differential ROS generation induced by N-EVs and M-EVs correlates with differential activation of the NF-κB pathway and subsequent variation in the expression of downstream pro-inflammatory cytokines, we evaluated the nuclear translocation of the NF-κB p65 subunit in HUVECs after 24 h of treatment with either N-EVs or M-EVs using IF staining. The results indicated that M-EV treatment significantly increased the nuclear translocation of NF-κB p65 compared to N-EV treatment (Figure 4A). Concurrently, the results of the ELISA demonstrated that both N-EVs and M-EVs significantly upregulated the expression of downstream pro-inflammatory cytokines, including TNF-α, IL-1β, and IL-6 (Figure 4B). Moreover, compared to N-EVs, M-EVs induced a significantly greater increase in the expression levels of these cytokines. Collectively, these findings suggest that EVs produced by *P. gingivalis* under sub-inhibitory concentrations of metronidazole treatment conditions potentiate the pro-inflammatory response in endothelial cells.

## 4. Discussion

Previous research has demonstrated that multiple factors influence the production and yield of *P. gingivalis* EVs, including the structural characteristics and modification status of lipid A, gingipains, peptidyl arginine deiminase (PPAD), autolysin, and the fimbrial subunit A (FimA) [9,17,24,25]. More recently, environmental conditions affecting bacterial growth—such as antibiotic exposure, pH, temperature, nutrient availability, and oxidative stress—have also been implicated in the regulation of EV formation [26]. Among these, antibiotics, particularly those targeting Gram-negative bacteria, have garnered increasing attention for their role in modulating EV biogenesis. For instance, Kadurugamuwa et al. [27] reported that gentamicin at four times the minimum inhibitory concentration (MIC) destabilized the outer membrane of *Pseudomonas aeruginosa* (*P. aeruginosa*), resulting in a 3-to-5-fold increase in EV release. Metronidazole, a nitroimidazole antibiotic widely employed in clinical settings due to its efficacy against anaerobic bacteria, has similarly been shown to influence EV secretion. Ribeiro de Freitas et al. [28] demonstrated that sub-inhibitory concentrations levels of metronidazole significantly enhanced EV secretion in *Bacteroides fragilis* (*B. fragilis*). In the present study, we provide the first evidence that sub-inhibitory concentrations of metronidazole induce an approximately 2.3-fold increase in EV release from *P. gingivalis*. Although this phenomenon has been observed previously and herein, the precise molecular mechanisms underlying metronidazole-induced EV secretion in *P. gingivalis* remain unclear. Notably, Ribeiro de Freitas et al. [28] highlighted a strong association between increased *B. fragilis* EV secretion and bacterial stress responses. Furthermore, our findings indicate that sub-inhibitory concentrations of metronidazole exposure lead to a reduction in *P. gingivalis* cell size and a morphological shift from elongated rods to elliptical forms. This morphological alteration likely represents a direct response to metronidazole-induced stress, potentially involving the SOS response and oxidative stress pathways [29]. Such changes may facilitate EV biogenesis by decreasing outer membrane stability and modifying its composition, thereby promoting EV outgrowth and secretion.

Lipid A of *P. gingivalis* constitutes a principal toxic component of its LPS and plays a critical role in the pathogenesis of periodontal disease and its associated systemic disorders [17,30]. Lipid A activates the NF-κB signaling pathway through binding to Toll-like receptors (TLRs) on host cell surfaces, thereby inducing excessive release of pro-inflammatory cytokines and chemokines, which leads to persistent localized periodontal inflammation [31]. Upon translocation into the circulation via disrupted periodontal epithelium, lipid A can provoke systemic low-grade inflammation, which is closely linked to As, diabetes, and Alzheimer’s disease [17,31]. The gingipains of *P. gingivalis*, primarily RgpA/B and Kgp, are key virulence factors belonging to the cysteine protease family; they play a central role in host tissue destruction, immune evasion, and facilitation of bacterial colonization and biofilm formation [9,10]. In the present study, *P. gingivalis* EVs were found to be enriched in lipid A, RgpA, and Kgp. Notably, EVs produced under sub-inhibitory concentrations of metronidazole exposure exhibited increased abundance of lipid A, Kgp, and RgpA, potentially enhancing their invasive capacity against host cells. Kim et al. [32] demonstrated that EVs induced by 1/4 MIC ceftazidime from *Burkholderia cepacia* (*B. cepacia*) augmented host cytotoxicity and pro-inflammatory responses. Similarly, Ribeiro de Freitas et al. [28] reported that sub-inhibitory concentrations of metronidazole significantly increased secretion of *B. fragilis* EVs, which exacerbated inflammatory responses by activating host immune cells and markedly promoting TNF-α and IL-6 secretion. Consistent with these findings, the in vitro experiments conducted in this study provide the first evidence that sub-inhibitory concentrations of metronidazole-induced *P. gingivalis* EVs exhibit significantly enhanced cytotoxicity toward HUVECs. Mechanistically, compared to naturally derived *P. gingivalis* EVs, those induced by sub-inhibitory concentrations of metronidazole elicited significantly elevated levels of mtROS and total cellular ROS generation. It is well established that ROS can activate IκB kinase (IKK), resulting in the phosphorylation and ubiquitin-mediated degradation of the inhibitory protein IκBα. This degradation releases NF-κB, which is otherwise sequestered in the cytoplasm in an inactive complex with IκBα, thereby permitting its translocation into the nucleus. Once in the nucleus, NF-κB promotes a marked upregulation in the expression of pro-inflammatory cytokines, including TNF-α, IL-1β, and IL-6 [22,23]. This study further demonstrated that sub-inhibitory concentrations of metronidazole-induced *P. gingivalis* EVs robustly induced nuclear translocation of the NF-κB p65 subunit and increased expression of its downstream pro-inflammatory cytokines TNF-α, IL-6, and IL-1β. The overexpression of these cytokines may contribute to atherosclerotic plaque formation and instability by compromising vascular endothelial barrier integrity, promoting lipid deposition, and facilitating platelet aggregation, thereby elevating the risk of CVD [23,33]. Consequently, *P. gingivalis* EVs induce endothelial dysfunction via the ROS/NF-κB signaling pathway, with sub-inhibitory concentrations of metronidazole-induced EVs exacerbating endothelial damage through this mechanism. This critical finding provides a novel mechanistic explanation for the suboptimal therapeutic efficacy of antibiotics observed clinically in periodontitis patients with comorbid CVD. We hypothesize that under the selective pressure of sub-inhibitory concentrations of antibiotics, periodontal pathogens secrete EVs with enhanced pathogenicity. These EVs persistently disrupt the vascular endothelial barrier and, by amplifying systemic inflammation, accelerate the progression of vascular pathology.

## 5. Conclusions and Future Perspectives

Although this study demonstrates that sub-inhibitory concentrations of metronidazole enhance *P. gingivalis* EV production and enrichment of core virulence factors, leading to increased endothelial cytotoxicity, oxidative stress, and inflammation in HUVECs, several mechanistic aspects remain unresolved. Key limitations include the uncharacterized molecular basis for metronidazole-induced Es biogenesis and the incomplete profiling of EV pathogenic components beyond three virulence factors. Furthermore, the specific EV components driving endothelial dysfunction and their potential synergies require elucidation. Future research should aim to characterize the principal effector molecules and confirm the underlying mechanisms in vivo. Nevertheless, these findings provide a crucial experimental basis for understanding the persistence of vascular inflammation despite low viable *P. gingivalis* abundance in atherosclerotic plaques and the limited efficacy of antibiotics in reducing adverse cardiovascular events in periodontitis patients.

Furthermore, it should be noted that in clinical practice, metronidazole is often administered in combination with antibiotics such as amoxicillin to broaden the antimicrobial spectrum and achieve synergistic effects. Our study, however, only investigated the impact of metronidazole monotherapy. Future research warrants an in-depth exploration of whether the secretion, protein composition, and function of *P. gingivalis* EVs would undergo more complex alterations under the combined influence of metronidazole and amoxicillin or other antibiotics. Would such changes simply amplify or diminish the effects observed with single-drug treatment, or would they lead to entirely novel patterns—synergistic or antagonistic—driven by drug interactions? Elucidating this issue is of significant importance for more accurately assessing the indirect impact of clinical combination regimens on the pathogenic potential of periodontal pathogens.

## Data Availability

The original contributions presented in this study are included in this article/Supplementary Materials. Further inquiries can be directed to the corresponding author.

## Supplementary Materials

The following supporting information can be downloaded at: https://www.mdpi.com/article/10.3390/microorganisms14010025/s1, Figure S1: (A) Revival and culture of *P. gingivalis* on blood agar plates. (B) Growth curve analysis of *P. gingivalis* (three repeated averages). (C) Coomassie brilliant blue staining of *P. gingivalis* and EVs. Figure S2: (A) Results of visual inspection for the inhibitory effect of metronidazole against *P. gingivalis*. (B) Protein concentration of N/M/2M-EVs quantified by BCA assay. (C) SEM images showing the surface morphology of *P. gingivalis* (scale bar, 1 μm). Data in panels B was analyzed by one-way ANOVA and are presented as the mean ± SD from three independent experiments. Statistical significance is indicated as follows: ns = not significant; *** *p* < 0.001. 2M-EVs: 4 μg/mL metronidazole. Table S1: Absorbance values of different concentrations of metronidazole solutions against *Porphyromonas gingivalis*.
