# Supplementary material for: Sub-Inhibitory Concentrations of Metronidazole Enhance Production, Virulence Factor Loading, and Endothelial Cytotoxicity of Porphyromonas gingivalis Extracellular Vesicles
Source: Microorganisms. 2025 Dec 21;14(1):25. doi: 10.3390/microorganisms14010025 (PMC12843824; doi:10.3390/microorganisms14010025)
Supplement: Supplementary file 1 [file microorganisms-14-00025-s001.zip › Supplementary figure.pptx]

## Slide 1
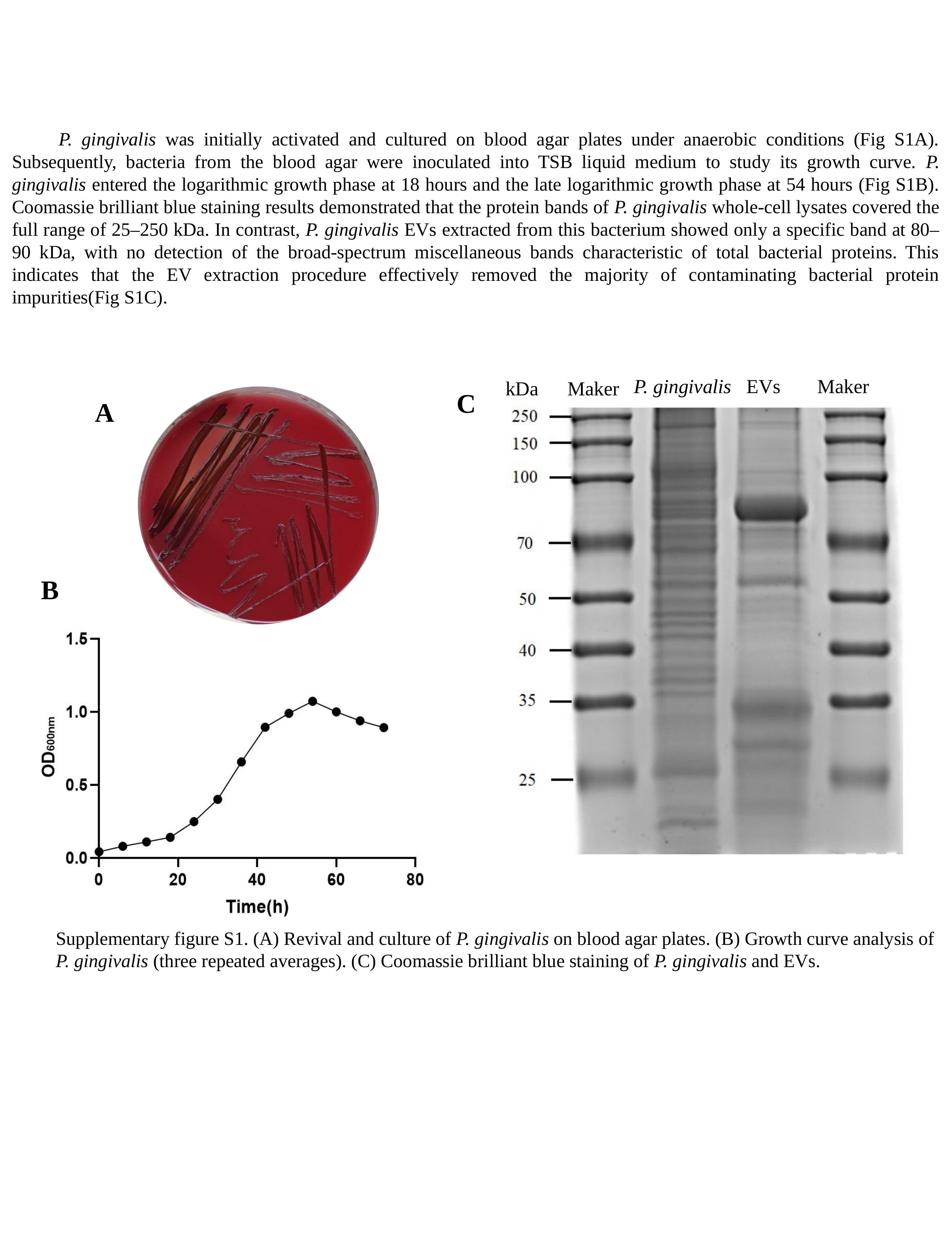

P. gingivalis was initially activated and cultured on blood agar plates under anaerobic conditions (Fig S1A). Subsequently, bacteria from the blood agar were inoculated into TSB liquid medium to study its growth curve. P. gingivalis entered the logarithmic growth phase at 18 hours and the late logarithmic growth phase at 54 hours (Fig S1B). Coomassie brilliant blue staining results demonstrated that the protein bands of P. gingivalis whole-cell lysates covered the full range of 25–250 kDa. In contrast, P. gingivalis EVs extracted from this bacterium showed only a specific band at 80–90 kDa, with no detection of the broad-spectrum miscellaneous bands characteristic of total bacterial proteins. This indicates that the EV extraction procedure effectively removed the majority of contaminating bacterial protein impurities(Fig S1C).
P. gingivalis
EVs
Maker
kDa
Maker
C
A
B
Supplementary figure S1. (A) Revival and culture of P. gingivalis on blood agar plates. (B) Growth curve analysis of P. gingivalis (three repeated averages). (C) Coomassie brilliant blue staining of P. gingivalis and EVs.

## Slide 2
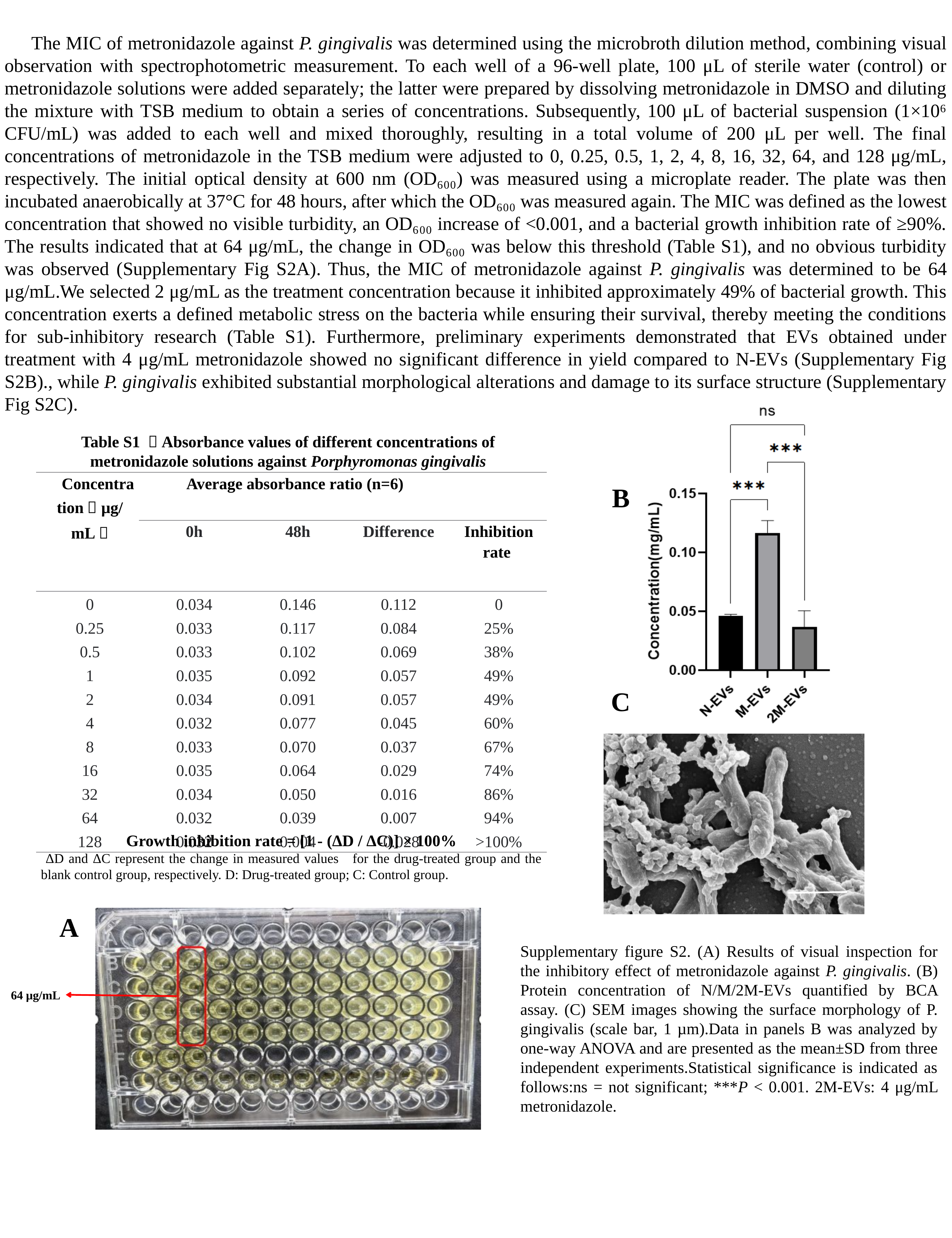

The MIC of metronidazole against P. gingivalis was determined using the microbroth dilution method, combining visual observation with spectrophotometric measurement. To each well of a 96-well plate, 100 μL of sterile water (control) or metronidazole solutions were added separately; the latter were prepared by dissolving metronidazole in DMSO and diluting the mixture with TSB medium to obtain a series of concentrations. Subsequently, 100 μL of bacterial suspension (1×10⁶ CFU/mL) was added to each well and mixed thoroughly, resulting in a total volume of 200 μL per well. The final concentrations of metronidazole in the TSB medium were adjusted to 0, 0.25, 0.5, 1, 2, 4, 8, 16, 32, 64, and 128 μg/mL, respectively. The initial optical density at 600 nm (OD₆₀₀) was measured using a microplate reader. The plate was then incubated anaerobically at 37°C for 48 hours, after which the OD₆₀₀ was measured again. The MIC was defined as the lowest concentration that showed no visible turbidity, an OD₆₀₀ increase of <0.001, and a bacterial growth inhibition rate of ≥90%. The results indicated that at 64 μg/mL, the change in OD₆₀₀ was below this threshold (Table S1), and no obvious turbidity was observed (Supplementary Fig S2A). Thus, the MIC of metronidazole against P. gingivalis was determined to be 64 μg/mL.We selected 2 μg/mL as the treatment concentration because it inhibited approximately 49% of bacterial growth. This concentration exerts a defined metabolic stress on the bacteria while ensuring their survival, thereby meeting the conditions for sub-inhibitory research (Table S1). Furthermore, preliminary experiments demonstrated that EVs obtained under treatment with 4 μg/mL metronidazole showed no significant difference in yield compared to N-EVs (Supplementary Fig S2B)., while P. gingivalis exhibited substantial morphological alterations and damage to its surface structure (Supplementary Fig S2C).
Table S1 ｜Absorbance values of different concentrations of metronidazole solutions against Porphyromonas gingivalis
| Concentration（μg/mL） | Average absorbance ratio (n=6) | | | |
| --- | --- | --- | --- | --- |
| | 0h | 48h | Difference | Inhibition rate |
| 0 | 0.034 | 0.146 | 0.112 | 0 |
| 0.25 | 0.033 | 0.117 | 0.084 | 25% |
| 0.5 | 0.033 | 0.102 | 0.069 | 38% |
| 1 | 0.035 | 0.092 | 0.057 | 49% |
| 2 | 0.034 | 0.091 | 0.057 | 49% |
| 4 | 0.032 | 0.077 | 0.045 | 60% |
| 8 | 0.033 | 0.070 | 0.037 | 67% |
| 16 | 0.035 | 0.064 | 0.029 | 74% |
| 32 | 0.034 | 0.050 | 0.016 | 86% |
| 64 | 0.032 | 0.039 | 0.007 | 94% |
| 128 | 0.032 | 0.004 | -0.028 | >100% |
B
C
Growth inhibition rate = [1 - (ΔD / ΔC)] × 100%
 ΔD and ΔC represent the change in measured values for the drug-treated group and the blank control group, respectively. D: Drug-treated group; C: Control group.
A
Supplementary figure S2. (A) Results of visual inspection for the inhibitory effect of metronidazole against P. gingivalis. (B) Protein concentration of N/M/2M-EVs quantified by BCA assay. (C) SEM images showing the surface morphology of P. gingivalis (scale bar, 1 µm).Data in panels B was analyzed by one-way ANOVA and are presented as the mean±SD from three independent experiments.Statistical significance is indicated as follows:ns = not significant; ***P < 0.001. 2M-EVs: 4 μg/mL metronidazole.
64 μg/mL
